# Strain‐Driven Bidirectional Spin Orientation Control in Epitaxial High Entropy Oxide Films

**DOI:** 10.1002/advs.202304038

**Published:** 2023-07-28

**Authors:** Zhibo Zhao, Arun Kumar Jaiswal, Di Wang, Vanessa Wollersen, Zhengyu Xiao, Gajanan Pradhan, Federica Celegato, Paola Tiberto, Maria Szymczak, Juliusz Dabrowa, Moaz Waqar, Dirk Fuchs, Xiaoqing Pan, Horst Hahn, Robert Kruk, Abhishek Sarkar

**Affiliations:** ^1^ Institute of Nanotechnology Karlsruhe Institute of Technology Eggenstein‐Leopoldshafen 76344 Karlsruhe Germany; ^2^ KIT‐TUD‐Joint Research Laboratory Nanomaterials Technical University Darmstadt 64287 Darmstadt Germany; ^3^ Institute for Quantum Materials and Technologies Karlsruhe Institute of Technology Eggenstein‐Leopoldshafen 76344 Karlsruhe Germany; ^4^ Karlsruhe Nano Micro Facility (KNMFi) Karlsruhe Institute of Technology 76131 Karlsruhe Germany; ^5^ Key Laboratory of Magnetic Molecules and Magnetic Information Materials of Ministry of Education School of Chemistry and Materials Science Shanxi Normal University Taiyuan 030031 China; ^6^ Advanced Materials and Life Science Divisions Istituto Nazionale di Ricerca Metrologica (INRiM) Turin 10135 Italy; ^7^ AGH University of Science and Technology Faculty of Materials Science and Ceramics al. Mickiewicza 30 Kraków 30‐059 Poland; ^8^ Department of Materials Science and Engineering University of California Irvine CA 92697 USA; ^9^ Department of Physics and Astronomy University of California Irvine CA 92697 USA; ^10^ Irvine Materials Research Institute University of California Irvine CA 92697 USA; ^11^ School of Sustainable Chemical, Biological and Materials Engineering The University of Oklahoma Norman OK 73019 USA

**Keywords:** dual‐phase coexistence, high entropy oxides, interfacial segregation, perpendicular magnetic anisotropy, strain‐engineering

## Abstract

High entropy oxides (HEOs), based on the incorporation of multiple‐principal cations into the crystal lattice, offer the possibility to explore previously inaccessible oxide compositions and unconventional properties. Here it is demonstrated that despite the chemical complexity of HEOs external stimuli, such as epitaxial strain, can selectively stabilize certain magneto‐electronic states. Epitaxial (Co_0.2_Cr_0.2_Fe_0.2_Mn_0.2_Ni_0.2_)_3_O_4_‐HEO thin films are grown in three different strain states: tensile, compressive, and relaxed. A unique coexistence of rocksalt and spinel‐HEO phases, which are fully coherent with no detectable chemical segregation, is revealed by transmission electron microscopy. This dual‐phase coexistence appears as a universal phenomenon in (Co_0.2_Cr_0.2_Fe_0.2_Mn_0.2_Ni_0.2_)_3_O_4_ epitaxial films. Prominent changes in the magnetic anisotropy and domain structure highlight the strain‐induced bidirectional control of magnetic properties in HEOs. When the films are relaxed, their magnetization behavior is isotropic, similar to that of bulk materials. However, under tensile strain, the hardness of the out‐of‐plane (OOP) axis increases significantly. On the other hand, compressive straining results in an easy OOP magnetization and a maze‐like magnetic domain structure, indicating the perpendicular magnetic anisotropy. Generally, this study emphasizes the adaptability of the high entropy design strategy, which, when combined with coherent strain engineering, opens additional prospects for fine‐tuning properties in oxides.

## Graphical abstract

The research presents a novel method that combines the concept of high entropy (HE) design with coherent epitaxial strain engineering to manipulate spin reorientation in ferrimagnetic HE oxides (HEOs). The authors demonstrate that epitaxial strain can selectively stabilize specific magnetoelectronic states, despite the extreme local chemical and bonding heterogeneity of HEOs.

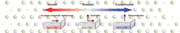

## 1 Introduction

Metal oxides with competing or nearly degenerate physical ground states often exhibit complex and fascinating physical properties that are a result of the interplay between different electronic, spin, and lattice degrees of freedom.^[^ ^1^ ^]^ In such cases, strain engineering offers a powerful tool for selective stabilization of the desired physical state by providing precise control over the structural distortions and defects in the material.^[^ ^2^ ^]^ This approach has been successfully utilized in magnetic oxides to enhance existing functionalities or to create entirely new ones.^[^ ^2^, ^3^, ^4^ ^]^ The newly introduced class of high entropy oxides (HEOs),^[^ ^5^, ^6^, ^7^, ^8^ ^]^ due to their exceptional chemical complexity, provides a natural setting for the competition/coexistence of magneto‐electronic interactions. HEOs are compositional complex single‐phase solid solutions that are based on the incorporation of multiple cations (typically ≥ 5) in near‐equal concentrations to a given cation sub‐lattice.^[^ ^9^, ^10^, ^11^ ^]^ This strategy, which relies on the enhancement and control of configurational disorder, offers the possibility to explore the unraveled composition space close to the center of multinary oxide phase diagrams with anticipation of potentially unique properties.^[^ ^11^, ^13^, ^14^ ^]^ Consequently, the field of HEOs has rapidly grown to include several compositions and crystallographic structures such as transition metal (TM)‐based rocksalt‐HEOs and spinel‐HEOs, rare‐earth (RE)‐based fluorite‐HEOs, and RE‐TM‐based perovskite‐HEOs.^[^ ^6^, ^12^, ^13^, ^14^ ^]^ Likewise, multiple studies showcase the wide variety of unique and often improved functionalities exhibited by the HEOs compared to the conventional oxide systems such as enhanced electrochemical cyclic stability, high catalytic activity, better thermal insulation, and tailorable magneto‐electronic properties.^[^ ^9^, ^15^, ^16^, ^17^, ^18^, ^19^, ^20^, ^21^ ^]^

Although there are limited research works on strain engineering in HEOs, the available reports suggest promising opportunities for further exploration of structure‐property relationships.^[^ ^19^, ^20^, ^22^ ^]^ Conventionally, to achieve a large magnitude of coherent elastic strain in oxides, thin films are epitaxially grown on a substrate, that is, in such a way that the crystal structure of the oxide layer is aligned with that of the substrate. Strain in the oxide film, of magnitudes that far exceeds what can be accommodated in the bulk, stems from the substrate‐film lattice constant differences.^[^ ^23^ ^]^ Such strain has been applied to control spin orientation in materials, induce interfacial magnetism in non‐magnetic oxides, and modify ferromagnetic and superconducting transition temperatures.^[^ ^4^, ^23^, ^24^, ^25^ ^]^ The ability to manipulate spin reorientation through magneto‐crystalline anisotropy in spinel oxides is a technologically relevant attribute that is particularly sensitive to induced strain.^[^ ^26^, ^27^ ^]^ The magneto‐crystalline anisotropy arises from a combination of spin‐orbit coupling (SOC) (between cation d‐orbital electron spin (*S*) and orbital moment (*L*_orb_)) and electrostatic interactions of d‐electrons with the crystal field.^[^ ^28^ ^]^ In the compositionally complex spinel‐HEOs this spin‐lattice coupling is expected to exhibit significant local variations, depending on the spin‐electronic state of the surrounding ions and the local distortions of the crystal lattice. In this case study, we have combined the principles of coherent epitaxial straining and a HE‐based approach, which inherently involves significant incoherent lattice distortions, to engineer the magnetic properties of a spinel‐HEO.

A well‐known TM‐based spinel‐HEO with a bulk composition equivalent to (Co_0.2_Cr_0.2_Fe_0.2_Mn_0.2_Ni_0.2_)_3_O_4_ is used as the model system. It should be noted that unlike other HEOs, where the configurational entropy is known to govern the random cationic occupancy, (Co_0.2_Cr_0.2_Fe_0.2_Mn_0.2_Ni_0.2_)_3_O_4_ (hereafter termed as HEO, irrespective of their cation distribution) shows a strong degree enthalpy‐mediated (crystal field stabilization energy) ionic ordering, (Co_0.6_Fe_0.4_)(Cr_0.3_Fe_0.1_Mn_0.3_Ni_0.3_)_2_O_4_.^[^ ^29^, ^30^ ^]^ The HEO composition in its bulk form is known to exhibit a Néel type ferrimagnetic ordering with *T*_c_ ≈ 420 K.^[^ ^31^ ^]^ In this work, epitaxial HEO thin films of varying thicknesses are deposited on three different single crystal substrates with (001) orientation, perovskite‐SrTiO_3_ (STO), rocksalt‐MgO, and spinel‐MgAl_2_O_4_ (MAO), that induce different kinds of strain (relaxed vs tensile vs compressive) in the HEO. Detailed structural investigations have been performed to evaluate the strain states and accompanying defects at the interfaces. The results of magnetometry measurements and investigations of the domain structure underline the potential of combining coherent strain engineering with local chemistry‐driven incoherent distortions to manipulate various magnetic characteristics, particularly magnetic anisotropy, in chemically disordered HEO materials.

## 2 Results and Discussion

In order to study the effects of different types of straining (tensile, compressive, or relaxed) and the degree of strain on the structure and magnetic properties of HEO, thin films are simultaneously deposited on STO, MgO, and MAO to ensure identical deposition conditions. The surface morphology, crystallinity, and epitaxy are characterized using atomic force microscopy (AFM), high‐resolution X‐ray diffraction (XRD), X‐ray reflectivity (XRR), and reciprocal space mapping (RSM). To gain insight into the structure and the interface at the local scale, high‐resolution transmission electron microscopy (TEM) is employed. The global and local magnetic characteristics are probed using a superconducting quantum interference device (SQUID) magnetometer and magnetic force microscopy (MFM), respectively.

### 2.1 Structural Features of HEO Films on MAO, MgO, and STO

#### 2.1.1 Global Structural Features

**Figure 1:**
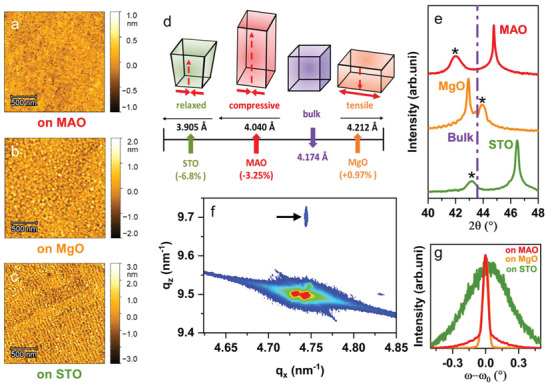
AFM images of 20 nm HEO thin films on MAO (a), STO (b), and MgO (c) substrates. The root‐mean‐square surface roughness of the HEO film deposited on MAO, MgO, and STO, is 0.145, 0.350, and 0.647 nm, respectively. Schematic of lattice parameters and mismatch of HEO on various substrates (d) and corresponding observed strain states. e) HRXRD of out‐of‐plane diffraction of 20 nm HEO on various substrates. f) Reciprocal space map of 20 nm HEO thin film on MgO substrate, the arrow indicates the HEO reflection. g) ω‐scans of 20 nm HEO on various substrates.

The AFM micrographs in **Figure** **1**a–c illustrate the surface smoothness of the films with *R*_a_ (root‐mean‐square surface roughness) of 0.145, 0.350, and 0.647 nm deposited on MAO, MgO, and STO, respectively. The analysis of the XRR data shows that the film thicknesses are close to 20 nm as shown in Figure S1, Supporting Information. The full range *θ*‐2*θ* XRD scans for the films deposited on STO, MgO, and MAO are also presented in Figure S1, Supporting Information. The presence of only (00*l*) reflections underscores the complete epitaxy and the phase purity of the HEO films deposited on all three substrates. The peak position of the HEO film in the vicinity of (002) reflection of STO and MgO, and (004) reflection of MAO, as shown in Figure 1e, is used for determining their out‐of‐plane (OOP) lattice parameter. The HEO in its bulk form (as is the case in the homemade ceramic target) has a lattice parameter of 8.348 Å. It should be noted that for comparison half of the lattice parameter of HEO (4.174 Å) and spinel‐MAO (4.040 Å) is used, which is equivalent to the lattice parameter of STO (3.905 Å) and MgO (4.212 Å) (Figure 1d). The corresponding lattice mismatch between the bulk HEO and the substrates is +0.97%, −3.25%, and −6.8% for MgO, MAO, and STO, respectively, as shown in Figure 1d. Consequently, different types and degrees of epitaxial strain (or tetragonality) are induced in the HEO films when deposited on MgO, MAO, and STO. The film deposited on MgO exhibits an elongation of the in‐plane (IP) lattice parameter (*a*) and corresponding compression of the OOP parameter (*c*). Thus, HEO film deposited on MgO is tensile strained with the actual degree of strain (*ε*) of +0.97%, featuring perfect cube‐one‐cube growth as indicated by the RSM and TEM results, which are discussed later in detail. *ε* is defined as the difference between the IP lattice parameter of the HEO thin film and the lattice parameter of the bulk HEO. HEO deposited on the MAO exhibits *ε* = −3.19%, indicating compressive straining. The film deposited on STO is almost completely relaxed, given the large lattice mismatch with the substrate, with a minor degree of residual compressive *ε* of −0.4%. Further information about *ε* can be obtained from RSM and rocking curve measurements (*ω*‐scans) as shown in Figure 1f,g. The *ω*‐scan of HEO on MgO exhibits a narrow full width of half maxima (FWHM) of 0.078° (Figure 1g), which along with the clear thickness oscillations observed in the XRD pattern (Figure 1e), highlights the superior epitaxial quality of the film. RSM for HEO on MgO (in Figure 1f) shows that the sample and the substrate have identical *q*_x_ values, thus, further confirming the highly coherent tensile strain in the sample. In contrast, the broad FWHM = 0.47° in the case of HEO on STO affirms the relaxed nature of the film. The *ω*‐scans of HEO on MAO exhibit two components, one with a broad FWHM of ≈0.3° and another one with a narrow FWHM of ≈0.077° (Figure 1g). The narrow one indicates the superior epitaxial quality of the film with negligible mosaicity, while the broader one shows a significant spread of mosaicity in certain regions of the epitaxial film.

**Figure 2:**
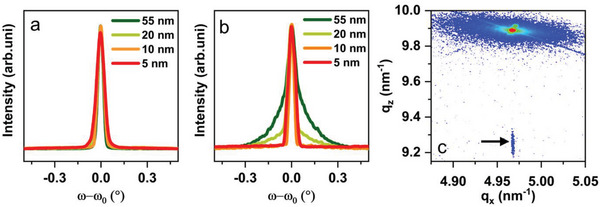
Thickness dependence of HEO thin films under tensile and compressive strain. ω‐scans of 55, 20, 10, and 5 nm HEO on MgO (a) and MAO (b). RSM of 10 nm HEO on MAO.

In order to gain more insight into the evolution of mosaicity and imperfections in the crystal, compressively strained HEO films of varying thicknesses (5, 10, 20, and 55 nm) were deposited on MAO. Likewise, for comparison, tensile strained films with similar thicknesses were deposited on MgO. The XRD data, presented in Figure S2, Supporting Information, indicate that all the HEO films on both MgO and MAO are epitaxial. The *ω*‐scans, indicative of the crystal quality, are shown in **Figure** **2**. In the case of MgO (Figure 2a), irrespective of the thickness, all the films showed high epitaxial quality with FWHM <0.08°. However, a thickness‐dependent variation in the FWHM and (more precisely) amount of the mosaic components, can be observed in the compressive strained MAO films. The films with the lower thickness (5 and 10 nm) have only a single component in *ω*‐scans with FWHM <0.08° (Figure 2b). This indicates that with decreasing thickness the compressive strained films on MAO achieve superior epitaxial quality with less mosaicity compared to the aforementioned 20 nm films. The superior epitaxial quality of the thinner film is also evident from RSM maps, shown in Figure 2c. The identical in‐plane position of the film and substrate indicates that the film is completely strained. Although the 20 nm film is also fully strained (see Section 2.1.2), larger mosaic components result in a significantly weaker signal in the RSM scan (Figure S3, Supporting Information). Likewise, the compressively strained film with the higher thickness (55 nm) shows an even increased amount of the broad highly mosaic FWHM component, which indicates the epitaxial quality of HEO deposited on MAO lowers with increasing thickness.

#### 2.1.2 Local Structural Features and Dual Phase Coexistence

**Figure 3:**
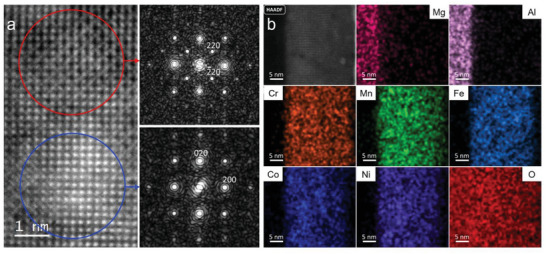
TEM analysis of 20 nm HEO on MAO substrate. High‐resolution HAADF‐STEM image of the 20 nm SHEO thin film on MAO (a), the red circle marks the spinel phase and the blue circle marks the rocksalt phase. A complete coherency between the spinel and rocksalt phases is evident. The right part of the panel (a) displays the fast Fourier transforms (FFTs) obtained from the marked regions in (a). HAADF‐STEM and corresponding elemental distribution maps (b) confirm the homogeneous distribution of the elements.

**Figure 4:**
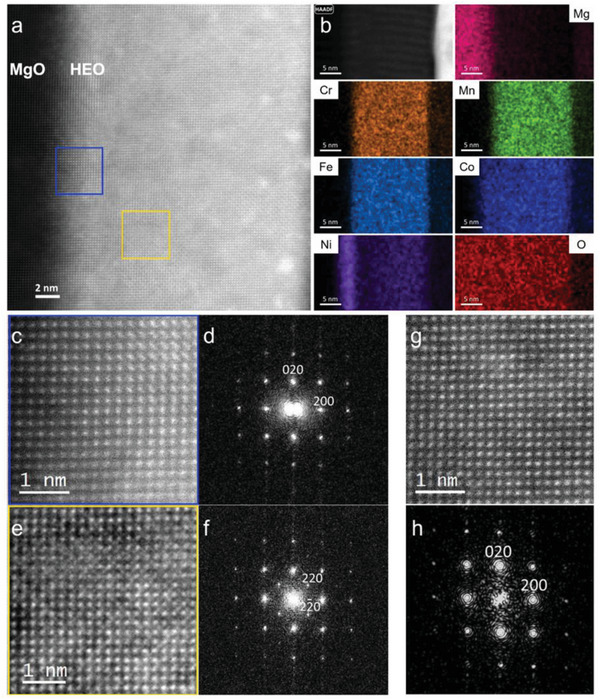
TEM analysis of 20 nm HEO on MgO substrate. High‐resolution HAADF‐STEM micrograph (a) indicates the fully epitaxial growth of HEO on MgO. The blue square on (a) along with (c,d) indicates a presence of an interfacial rocksalt phase (≈3 nm thick). Ni and Co enrichment at the interface (rocksalt layer) is evident from the elemental distribution maps (b). In the interior of the film (yellow square in (a)) both spinel‐HEO (e,f) and rocksalt (g,h) could be observed. Elemental distribution maps (b) confirm the homogeneous distribution of the elements at the interior of the film.

TEM along with energy dispersive spectroscopy (EDS) is used to gain deeper insight into the local structural feature, especially at the substrate‐film interfaces, of the 20 nm relaxed, compressive, and tensile strained HEO that are later used for the magnetic studies (**Figures** **3** and **4**). High‐resolution micrographs shown in Figure S4, Supporting Information, (MAO), and Figure 4a (MgO) reveal full epitaxy at the substrate‐film interfaces. This result is consistent with the aforementioned global structural results obtained from XRD. TEM micrograph of HEO on STO (Figure S5, Supporting Information) indicates the large lattice mismatch at the film‐substrate interface, however, regions of lattice coherency can still be overserved. In addition to the XRD findings, TEM analysis of several areas reveals that two distinct, fully coherent HEO phases coexist in all the HEO films: spinel and rocksalt.

First, we examine the coexistence of the dual phases in HEO films deposited on MAO. Their presence is quite prominent in the Figure 3a micrograph, where two neighboring regions show different atomic arrangements. Correspondingly, the fast Fourier transforms (FFTs) obtained from these regions are different. The region indicated by the red circle corresponds to the spinel phase showing additional super‐structure diffraction spots in the FFT, while the region marked by the blue circle belongs to the rocksalt phase. Importantly, Figure 3a reveals that the rocksalt and spinel phases are completely coherent without any interfacial boundary, indicating almost identical lattice spacing. Furthermore, the EDS maps (Figure 3b) exhibit no observable local scale elemental segregation. This implies that the rocksalt and spinel‐HEO phases are of very close compositions. Evidence for similar dual‐phase coexistence, without chemical segregation, is also observed in the case of relaxed films on STO (Figures S6 and S7, Supporting Information).

The coexistence of completely coherent spinel and rocksalt‐HEO phases without any distinguishable compositional fluctuations can as well be observed in the bulk of the tensile thin film deposited on MgO, that is, ≈3 nm away from the film‐substrate interface (Figure 4a,b,e–h). However, in addition, a 3 nm thick rocksalt phase enriched with Ni and Co can be observed at the film‐substrate interface of MgO (Figure 4a,c,d). This (Ni,Co)O‐rocksalt interfacial phase grows fully epitaxial on MgO, and HEO (spinel + rocksalt) further grows epitaxial on this interfacial rocksalt phase. The formation of this additional interfacial rocksalt phase in the case of MgO, which is absent in films on MAO and STO, is plausible because of the rocksalt structure of MgO. However, the reason for the formation of HEO phases, that is, rocksalt + spinel, in all the thin films, irrespective of the substrate, remains an open question. This result is rather surprising considering that such dissociation of the spinel‐HEO phase has not been reported in bulk.^[^ ^14^, ^29^ ^]^ However, a very recent study reports a similar dual‐phase existence in spinel‐HEO thin film grown on MAO.^[^ ^32^ ^]^ Hence, when deposited as an epitaxial thin film on any substrate (MgO, MAO, or STO), the formation or dissociation of the spinel‐HEO phase into rocksalt‐HEO and spinel‐HEO with close lattice constants and chemical compositions appears to be a universal phenomenon that is not otherwise observed in the bulk.

It should be noted that the exact ratio of the rocksalt‐HEO to spinel‐HEO phase that is observed in all the films (on MgO, MAO, or STO) is not easy to quantify. Nevertheless, we used O‐*K* edge electron energy loss spectra (EELS) analysis that provides an estimation of the spinel‐HEO and rocksalt‐HEO phases. The differentiation is done based on the hybridization of O 2p and transition metal 3d electrons, which is distinct for spinel and rocksalt. The details of the O‐*K* edge analysis are presented in Figure S8, Supporting Information. The data unambiguously show that the spinel‐HEO phase is the main phase in all cases.

The rocksalt (Fm‐3m) and spinel (Fd‐3m) structures are closely related, as they both share the same face‐centered cubic (fcc) oxygen frame, where only the sites occupied by the cations differ. In the rocksalt, all octahedral sites are evenly occupied by the cations. Conversely, in spinels, only a part of the octahedral sites is occupied while the rest are vacant, and instead, one‐eighth of tetrahedral sites are occupied. Spinel to rocksalt transitions can happen due to a variety of factors, of which the primary one is the lowering of the effective cationic oxidation state. If ideal stoichiometric conditions are met, cations in spinel oxides have an effective charge of 2.66+ compared to 2+ in rock salt oxides. Consequently, the introduction of hole doping in spinels tends to favor the rock salt structure. For instance, Li doping, either chemically or electrochemically, results in the transformation of spinel oxides to rocksalt structures.^[^ ^33^, ^34^ ^]^ The thin‐film deposition was carried out at an optimal O_2_ flow, which is similar to or higher than what has been used for depositing conventional spinel oxides with similar constituents.^[^ ^20^ ^]^ Furthermore, post‐annealing treatment of the deposited thin films in an air atmosphere (at 700 °C for 6 h) was performed to see if the rocksalt‐HEO phase disappears. TEM results confirm that the rocksalt‐HEO phase is present even after annealing (Figure S9, Supporting Information). These observations, to a large extent, rule out the fact that the rocksalt‐HEO phase stems from the synthesis conditions. Hence, the presence of the rocksalt‐HEO phase can plausibly be a result of the substrate‐induced straining, that somehow increases the preference for forming a rocksalt structure (maybe as a strain accommodation mechanism, which by the way is also relevant for STO) than a single spinel‐HEO phase. The factors that promote and stabilize the formation of the dual phase certainly remain an open question for future investigation. In any case, it seems like the spinel of (Co_0.2_Cr_0.2_Fe_0.2_Mn_0.2_Ni_0.2_)_3_O_4_ composition is an interesting system that in bulk shows a strong preferential occupation^[^ ^29^, ^30^ ^]^ while in thin films displays a lattice‐coherent phase dissociation. Hence, exploring this system in detail, especially the origin and mechanism related to the rocksalt‐HEO phase formation in (Co_0.2_Cr_0.2_Fe_0.2_Mn_0.2_Ni_0.2_)_3_O_4_ epitaxial thin films, can provide valuable insights into the thermodynamics of HEOs as a whole.

### 2.2 Magnetic Properties and Domain Structure of 20 nm Films

#### 2.2.1 Bulk Magnetic Properties

**Figure 5:**
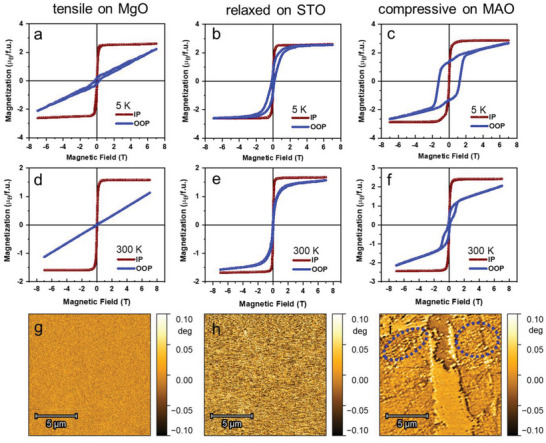
Global and local scale magnetic characterization of 20 nm HEO on various substrates. IP and OOP *M–H* loops at 5 K for HEO on MgO (a), STO (b), and MAO (c). 300 K *M–H* (d), (e), (f), and corresponding in‐plane MFM micrograph (g), (h), (j) for HEO on MgO, STO, and MAO substrates, respectively. The blue circles in (i) indicate the maze domain observed in the compressive strained films.

The temperature‐dependent magnetization measurement *(M–T*) curves are shown in Figure S10, Supporting Information, which indicate that the magnetic transition temperature is well‐above room temperature as is the case in the bulk HEO. It should be noted that the magnetic properties are primarily determined by the dominant ferrimagnetic spinel‐HEO phase, with insignificant contributions from the rocksalt phase, which is anticipated to be antiferromagnetic.^[^ ^32^ ^]^ The magnetic field‐dependent magnetization measurement (*M–H*) curves for all the samples measured at 300 and 5 K are shown in **Figure** **5**. A distinct change in the magnetic anisotropy as a function of straining can be observed by comparing the *M–H* curves obtained when the magnetic field is applied parallel (IP) and perpendicular (OOP) to the plane of the thin films. There are two prominent contributions to the magnetic anisotropy in the case of thin films: one is the shape anisotropy that results from the OOP demagnetizing field, and the other one is the magnetocrystalline anisotropy (MA).

**Figure 6:**
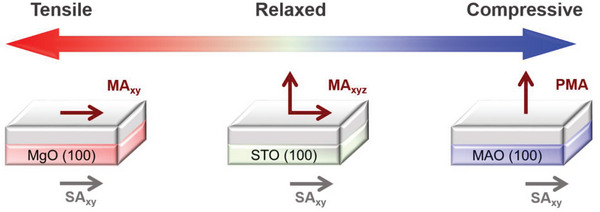
Schematic of strain‐driven bidirectional spin reorientation in epitaxial HEO thin films, indicating the hardness of the out‐of‐plane (OOP) upon tensile straining (MgO) and easiness of the OOP upon compressive strain (MAO). The relaxed film on STO behaves similar to the bulk, that is, isotropic, however, the shape anisotropy (SA) makes the in‐plane (*xy*) axis slightly easier compared to the OOP.

In all the cases (Figure 5), the IP *M–H* curves show a rather soft magnetic behavior, that is, saturation could be reached at low external magnetic fields (*H*_ext_ < 7 kOe) along with low coercivity, *H*_c_ (**Table** **1** and Figure S11, Supporting Information). In contrast, the slight disparities between the IP and OOP *M–H* curves (in Figure 5b,e) of the relaxed thin films on STO resemble a scenario similar to that of the bulk HEO. This indicates that the shape factor, although unavoidable, does not dominate the anisotropy landscape in these 20 nm epitaxial HEO thin films. The ratio of the magnetic remanence to saturation magnetization (*M*_R_ */M*_S_) is shown in Table 1. The results, especially at 5 K, indicating that there is a coexistence of minor components which has dominant perpendicular magnetic anisotropy (PMA) alongside the major component which is close to the bulk. This observation is schematically shown in **Figure** **6**. Despite the significant relaxation of the HEO film on STO, some degree of cube‐on‐cube growth and straining are expected at the interface, which is primarily compressive in nature (*ε* = −0.4%), as previously discussed. Thus, the PMA component observed in the hysteresis loop is likely to correspond to the minor strained region at the interface.

**Table 1:** The coercivity (*H*_C_) values (in kOe), saturated magnetization (*M*_S_) values (in *μ*_B_/f.u.), remnant magnetization (*M*_R_) values (in *μ*_B_/f.u.), and the ratio of *M*_S_/*M*_R_ for thin film HEO and bulk (at 5 K)

Strain	*H*_C_, OOP	*M*_S_, IP	*M*_R_, OOP	*M*_S_/*M*_R_	Ref.
Tensile (MgO)	2.5	2.54	0.18	100:7.3	This work
Relaxed (STO)	1.4	2.53	0.71	100:27	This work
Compressive (MAO)	12.5	2.87	1.38	100:50	This work
Bulk	0.23	1.73	0.17	100:10	[29]

In the case of the tensile strained films on MgO substrate, the OOP *M–H* curves at 5 and 300 K (Figure 4a,d) are significantly different from the IP, showing linear behavior up to *H*_ext_ = 70 kOe. The observation differs from the aforementioned relaxed scenario (HEO on STO), where the OOP *M–H* curves could be saturated at low fields, *H*_ext_ < 7 kOe. Given that the thickness (20 nm) of all the samples (on STO, MgO, and MAO) are similar, the anisotropy originating from the shape factor is practically identical. Hence, the distinct difference in the OOP magnetic behavior observed in the case of MgO is expected to originate solely from the strain effects. In‐plane stretching of the HEO film (and the accompanying compression of the OOP axis) appears to affect the MA, making the OOP direction a hard axis compared to the in‐plane one. The linear shape of the OOP curve with negligible remanence confirms that initially there is no component of magnetization along the OOP direction. Hence, almost the maximum of the available *H*_ext_ is used to rotate the spins from the IP to the OOP direction (Figure 6), but as soon as the field is removed, they rotate back to the easy axis, which in the case of the tensile straining lays in‐plane.

Figure 5c,f presents the IP and OOP *M–H* curves for the compressive strained HEO deposited on the MAO substrate. As mentioned earlier, the IP *M–H* curves for MAO are similar to that of the relaxed and tensile strained films on STO and MgO, respectively. However, the *OOP M–H* curves are remarkably different. The *M–H* curves collected at 5 K (Figure 5e) clearly show two magnetic components: one non‐saturating linear one, which is the minor component, and a saturated almost square loop, which is the predominant component. The square‐like *M–H* component with a high *H*_c_ and *M*_R_ */M*_S_ indicates that the compressive strained HEO has an easy axis in the OOP direction, similar to known systems exhibiting PMA. The room temperature *M–H* curve (Figure 5c) sheds more light on the magnetic behavior of HEO on MAO. There are noticeable three components in the magnetization curve. The non‐saturating linear one is identical to that observed at low temperatures. The square‐like PMA component observed at 5 K gives rise to the remaining two components at room temperature, one being very soft and the other relatively harder. The hard one, but with a lower *H*_c_ than observed at 5 K, can be naturally correlated to the PMA component. Increasing temperature, typically, weakens the magnetization as well as the MA. This explains the lower *H*_c_ at room temperature (Table 1) of the PMA component. On the other hand, faster softening of MA in some areas of the crystal may indicate magneto‐electronic inhomogeneity of the HEO spinel. Overall, based on magnetometry studies, it can be concluded that different types of strain have opposite effects on spin orientation, schematically shown in Figure 6. The compressively strained film on MAO shows PMA with OOP direction as the easy axis. The relaxed film on STO although showing a small degree of PMA, has largely isotropic character, that is, IP and OOP direction are mostly equivalent similar to the bulk HEO. In contrast, for the tensile strained MgO film, the OOP direction is clearly the hard axis. In the next section, we discuss MFM results that offer insights into the correlation between local and global magnetic properties.

#### 2.2.2 Local Magnetic Domain Structure

The MFM images of the tensile, relaxed, and compressively strained samples, acquired at room temperature in the IP magnetization remanence, are presented in Figure 5g–i. With the MFM being highly sensitive to the OOP magnetization component the dependency of the domain structure on the strain is evident. For the tensile strained HEO (on MgO), the MFM contrast is uniform without any OOP magnetization component, suggesting the presence of large in‐plane magnetic domains (Figure 5g). This is consistent with the soft IP behavior observed in the *M–H* curve, Figure 5a,d. The relaxed HEO on STO exhibits non‐uniform MFM contrasts (Figure 5h) reflecting a higher OOP component as compared to the tensile strained sample on MgO. This is also evident from the *M–H* curve that represents S‐shaped IP and OOP hysteresis loops indicating the magnetization switching by coherent rotation (see Figure 5e, blue curve). In contrast, the MFM image of the compressively strained film of HEO on MAO is quite different. A coexistence of two magnetic phases is visible. A typical maze domain structure on both sides is observed in Figure 5i and Figure S12, Supporting Information, which generally indicates the presence of an OOP magnetic anisotropy. In the middle, a wide band with plain and uniform contrast is observed similar to the sample deposited on MgO, representing a softer magnetic phase. The presence of two magnetic phases is also confirmed by the OOP hysteresis loop shown in Figure 5f where the magnetization reversal is characterized by a two‐step process. This further supports the conclusions made from the bulk magnetization data.

### 2.3 Discussion

Based on the experimental results, it appears that the type of strain imposed on the HEO epitaxial films has a significant effect on the spin orientation. Applying compressive strain to the film on the MAO substrate results in the spin orientation being perpendicular to the plane of the film, while the relaxed film on STO is magnetically isotropic. The tensile strain in the HEO film on the MgO substrate results in the IP direction being the easy axis. To uncover the mechanisms that drive these spin reorientations, several factors must be considered, including the arrangement of the ions across crystallographic sites, as well as their charge and spin states. Unfortunately, because of the highly complex chemical nature of HEO, this information cannot be readily obtained. Nonetheless, by examining the bulk counterpart of the studied HEO,^[^ ^29^ ^]^ some meaningful conclusions may be drawn. In the bulk compounds, the ion distribution, the charge, and the spin states were revealed in great detail by extensive X‐ray and X‐ray magnetic circular dichroism (XMCD) spectroscopy studies.^[^ ^29^ ^]^ Particularly, the analysis of the XMCD spectra offered precise information regarding the contribution of the cation's orbital moments to the overall moments.^[^ ^29^ ^]^ If one assumes that the ion distribution in the bulk and epitaxial films is similar, one can conclude that only Co^+2^, Co^+3^ ions occupying the tetrahedral and Ni^+2^ ions occupying octahedral sites have significant orbital moments. If we consider these facts, it can be inferred that mainly Ni and Co ions would be contributing to the magneto‐crystalline anisotropy. To simplify this, one can assume that their partially quenched orbital moment, denoted as *L*_orb_, would be coupled to the spin *S* of the d‐electrons through spin‐orbit interaction, which is proportional to *L*_orb_ × *S*. The next step involves the magneto‐elastic coupling between *L*_orb_ and the crystal field, which is necessary to induce spin rotation upon straining. Indeed, in recent studies,^[^ ^30^ ^]^ crystal field and magnetoelastic calculations were used to investigate strained spinels containing Co and Ni cations. The results indicate that applying compressive strain causes the easy axis to align OOP while applying tensile strain causes it to align IP. Thus, the strong magneto‐elastic response observed in this study implies that there are sufficient cooperative spin‐lattice interactions in HEO at the local level, resulting in a coherent rotation of the magnetization upon the strain.

## 3 Conclusions

This work demonstrates the use of coherent straining to control the degree of PMA in epitaxial spinel HEO thin films, with a bulk composition of (Co_0.2_Cr_0.2_Fe_0.2_Mn_0.2_Ni_0.2_)_3_O_4_. The strain states of the epitaxial HEO films, i.e., tensile, compressive, or relaxed, are tailored using three different epitaxial substrates, MgO, MAO, and STO, respectively. TEM studies reveal a unique coexistence of two fully coherent HEO phases in the thin films, namely a spinel and a rocksalt, with no discernible chemical fluctuations. This phenomenon, which is not observed in the bulk material of the same composition, appears to be a common occurrence when (Co_0.2_Cr_0.2_Fe_0.2_Mn_0.2_Ni_0.2_)_3_O_4_ is deposited as an epitaxial thin film. This observation implies that the free energy landscape of the HEOs has multiple energy minima that can be accessed by applying strain. The strain‐driven control of magnetic properties in HEO is manifested by the marked changes in magnetic anisotropy, as observed through magnetometry and spatially resolved MFM microscopy. The film grown on an STO substrate in a relaxed state displays almost isotropic magnetization behavior similar to bulk with no significant difference between in‐plane and OOP magnetization reversal. Under tensile strain, the hardness of the OOP axis significantly increases, and only the coherent spin‐rotation component from in‐plane to OOP is detected. Conversely, the HEO subjected to compressive strain shows an easy magnetization axis along the OOP direction, presenting a square‐like magnetization‐hysteresis (MH) loop and a maze domain structure that are characteristics of systems with PMA. To summarize, the magneto‐crystalline anisotropy of spinel‐HEO can be controlled via induced strain, despite their extreme variations in local bonding and magneto‐electronic heterogeneity. Thus, the present study demonstrates the numerous research possibilities for altering properties in oxide systems by using the high entropy method in conjunction with strain tuning of epitaxial thin films.

## 4 Experimental Section

### Materials Synthesis

HEO thin films were deposited on MgO (001), STO (001), and MAO (001) substrates by laser pulsed deposition technique (PLD). The material for the ceramic target of (Co_0.2_Cr_0.2_Fe_0.2_Mn_0.2_Ni_0.2_)_3_O_4_ was synthesized via the modified Pechini sol–gel method, as described in the Supporting Information. Prior to deposition, the STO (001) substrate was treated with acid followed by 980 °C annealing in air to form a TiO_2_ terminated surface. The commercial MgO, MAO, and treated STO substrates were all cleaned in isopropanol and deionized water in an ultrasonic bath each for 10 min to remove the particle and contaminants and dried by nitrogen blow. The thin film was deposited at 690 °C in 0.03 mbar oxygen pressure as optimal conditions. The KrF excimer laser with a wavelength of 248 nm was used for target ablation at a repetition rate of 5 Hz. The laser fluence was controlled at 1.55 J cm^−2^. The thickness of thin films was controlled by the given laser shots number: 500 shots (5 nm), 1000 shots (10 nm), 2000 shots (20 nm), and 6000 shots (55 nm). The target‐substrate distance was 25 mm. After deposition, the thin film was cooled to room temperature at 20 °C min^−1^.

### Characterization

Structural properties of the thin films were characterized by XRR, diffraction, and RSM using a Bruker D8 Davinci diffractometer. The surface morphology information of the S‐HEO thin film was scanned in tapping mode in the air by AFM of Bruker Dimension Icon. The scan size was 2 µm with 512 sample lines/image and a scan rate of 1 Hz.

TEM specimen of the thin film was prepared by a focused ion beam (FIB) (Strata dual beam, FEI Company). A double aberration‐corrected state‐of‐the art Themis Z (Thermo Fisher) TEM equipped with a Super‐X energy dispersive X‐Ray detector and Gatan GIF Continuum 970 HighRes + K3 IS camera (operated at 300 kV) were to used examine the specimens. STEM imaging of annealed sample was performed on JEOL JEM‐ARM300F aberration‐corrected microscope (operated at 300 kV). TEM and STEM micrographs were taken from different regions to confirm the large‐scale homogeneity of the samples.

Magnetic measurements at room temperature were performed on the powder samples using a Quantum Design MPMS3 SQUID in direct current (DC) mode. Magnetization versus temperature measurements were carried out at 200 Oe, using a zero‐field cooled (ZFC)‐field cooled (FC) sequence in the temperature range of 5–380 K. Magnetization versus magnetic field measurements were carried out at different temperatures between −7 and +7 T. Same measurements sequences were used for measuring the bare substrates, which were used for the background subtraction.

The MFM images were obtained by MultiModeTM SPM (Digital Instruments Veeco Metrology Group, Version 4.31ce). MESP magnetic tips having a resonant frequency of 75 kHz with Co/Cr reflective coating were used for acquiring the OOP magnetic signals.

## Conflict of Interest

The authors declare no conflict of interest.

## Author Contributions

Z.Z., R.K., and A.S. conceived and designed the experiments. Z.Z. and A.S. fabricated the samples and performed XRR, XRD, and rocking curve measurements. A.K.J. and D.F. performed the RSM measurements. D.W. and M.W. performed the TEM measurements and analysis. Z.Z. and Z.X. performed the SQUID and AFM measurements. V.W. prepared the FIB lamella. G.P., F.C., and P.T. performed the MFM scan. J.D. and M.S. provided the powders for the target preparation. A.S., R.K., X.P., and H.H. supervised the project. A.S., Z.Z., and R.K. wrote the manuscript with comments from other authors.

## Supporting information

Supporting Information

## Data Availability

The data that support the findings of this study are available from the corresponding author upon reasonable request.
