# Supplementary material for: Strain‐Driven Bidirectional Spin Orientation Control in Epitaxial High Entropy Oxide Films
Source: Adv Sci (Weinh). 2023 Jul 28;10(27):2304038. doi: 10.1002/advs.202304038 (PMC10520624; doi:10.1002/advs.202304038)
Supplement: Supplementary file 1 — Supporting Information [file ADVS-10-2304038-s001.pdf]

ADVANCED SCIENCE
Open Access

Supporting Information

for *Adv. Sci.*, DOI 10.1002/advs.202304038

Strain-Driven Bidirectional Spin Orientation Control in Epitaxial High Entropy Oxide Films

Zhibo Zhao, Arun Kumar Jaiswal, Di Wang, Vanessa Wollersen, Zhengyu Xiao, Gajanan Pradhan, Federica Celegato, Paola Tiberto, Maria Szymczak, Juliusz Dabrowa, Moaz Waqar, Dirk Fuchs, Xiaoqing Pan, Horst Hahn, Robert Kruk* and Abhishek Sarkar**

Strain-driven bidirectional spin orientation control in epitaxial high entropy oxide films

Zhibo Zhao[1,2], Arun Kumar Jaiswal[3], Di Wang[1,4,], Vanessa Wollersen[1,4], Zhengyu Xiao[1,5], Gajanan Pradhan[6], Federica Celegato[6], Paola Tiberto[6], Maria Szymczak[7], Juliusz Dabrowa[7], Moaz Waqar[8], Dirk Fuchs[3], Xiaoqing Pan[8,9,10], Horst Hahn[1,2,11], Robert Kruk[1,*], Abhishek Sarkar[1,2,*]*

[1]*Institute of Nanotechnology, Karlsruhe Institute of Technology, Eggenstein-Leopoldshafen 76344, Germany*

[2]*KIT-TUD-Joint Research Laboratory Nanomaterials, Technical University Darmstadt, Darmstadt 64287, Germany*

[3]*Institute of Quantum Materials and Technologies, Karlsruhe Institute of Technology, Eggenstein-Leopoldshafen 76344, Germany*

[4]*Karlsruhe Nano Micro Facility (KNMF), Karlsruhe Institute of Technology, 76131 Karlsruhe, Germany*

[5]*Key Laboratory of Magnetic Molecules and Magnetic Information Materials of Ministry of Education, School of Chemistry and Materials Science, Shanxi Normal University, Taiyuan 030031, China*

[6]*Italian National Institute of Metrology Research, Turin 10135, Italy*

[7]*AGH University of Science and Technology, Faculty of Materials Science and Ceramics, al. Mickiewicza 30, 30-059 Kraków, Poland*

[8]*Department of Materials Science and Engineering, University of California, Irvine, California 92697, USA*

[9]*Department of Physics and Astronomy, University of California, Irvine, California 92697, USA*

[10]*Irvine Materials Research Institute, University of California, Irvine, California 92697, USA*

[11]*The University of Oklahoma, Chemical, Biological and Materials Engineering, Norman, Oklahoma 73019, USA*

*Corresponding author: di.wang@kit.edu; robert.kruk@kit.edu; abhishek.sarkar@kit.edu

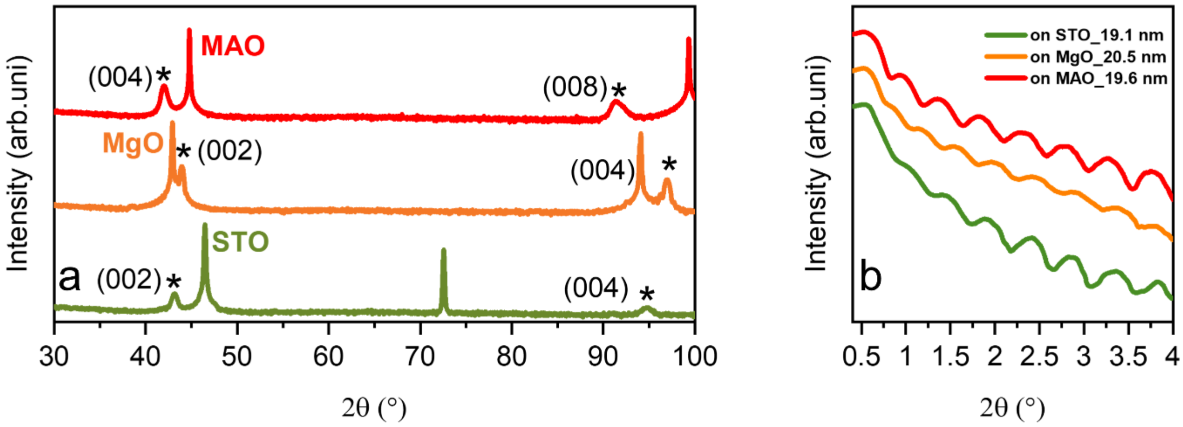

Figure S1. (a) Full range of HRXRD scan for 20 nm HEO thin films on MAO, MgO and STO substrates. (b) XRR of 2000 laser shots of HEO on three substrates.

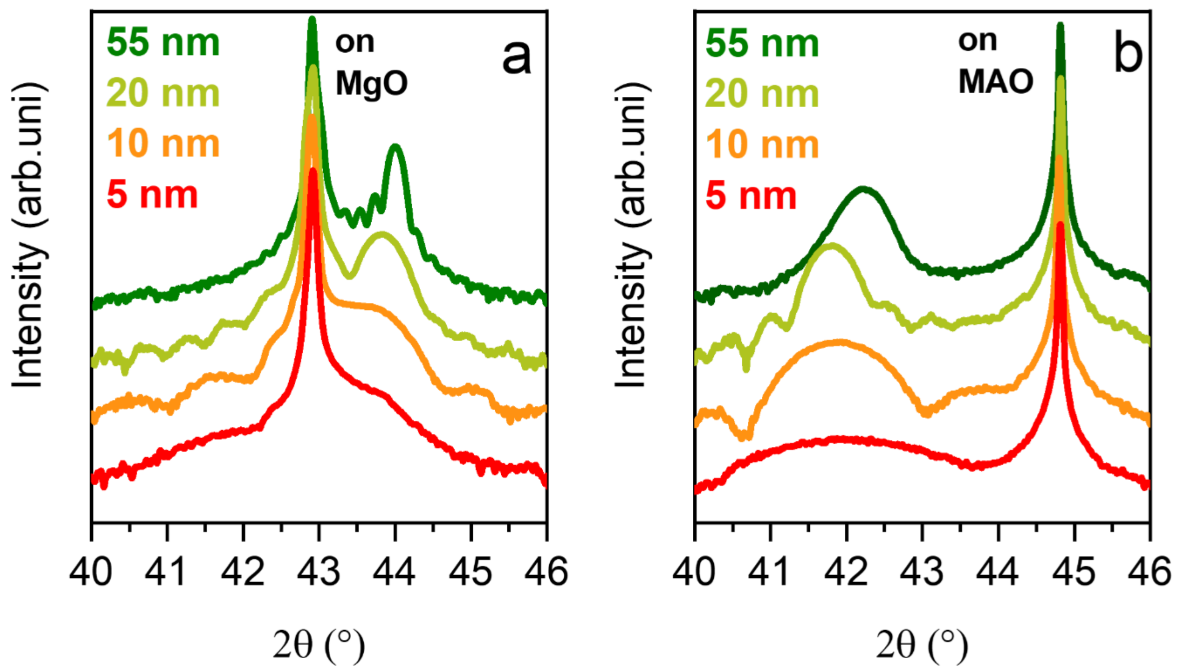

Figure S2. Thickness dependence of HEO thin films on various substrates: MgO (a) and MAO (b).

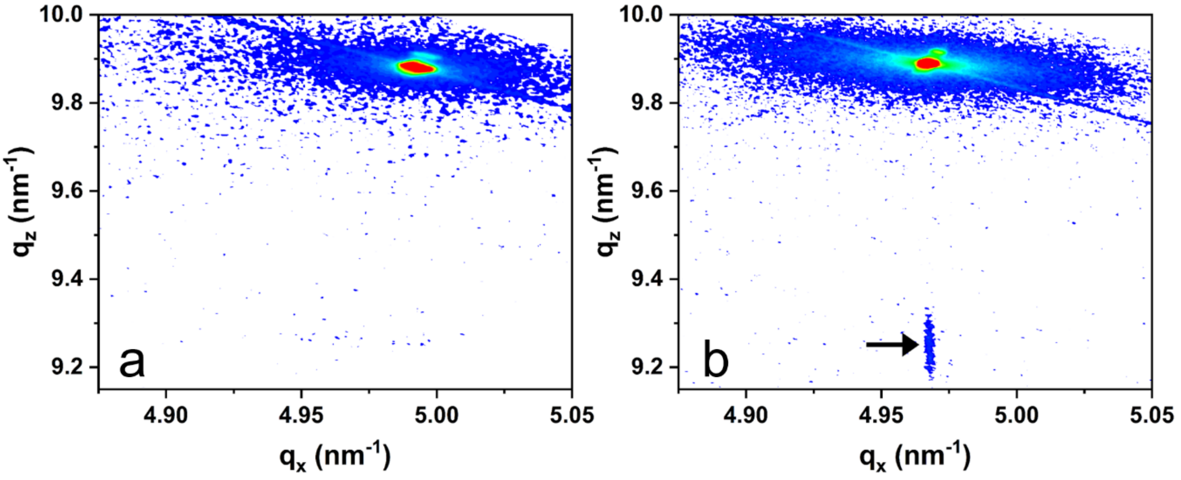

Figure S3. Thickness dependence of HEO thin films on MAO substrates: (a) 20 nm, (b) 10 nm.

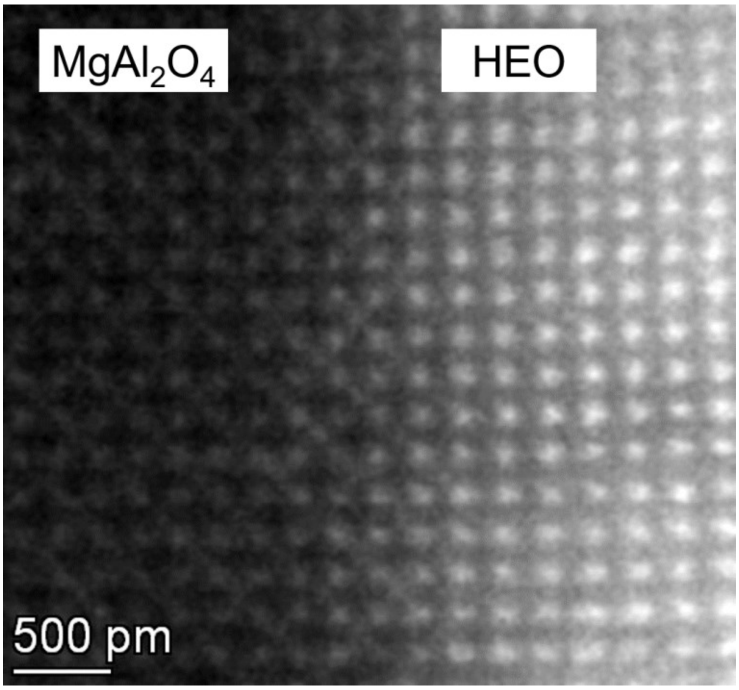

Figure S4. TEM micrograph of HEO deposited on MAO substrate. A complete lattice coherency between the substrate and film can be observed.

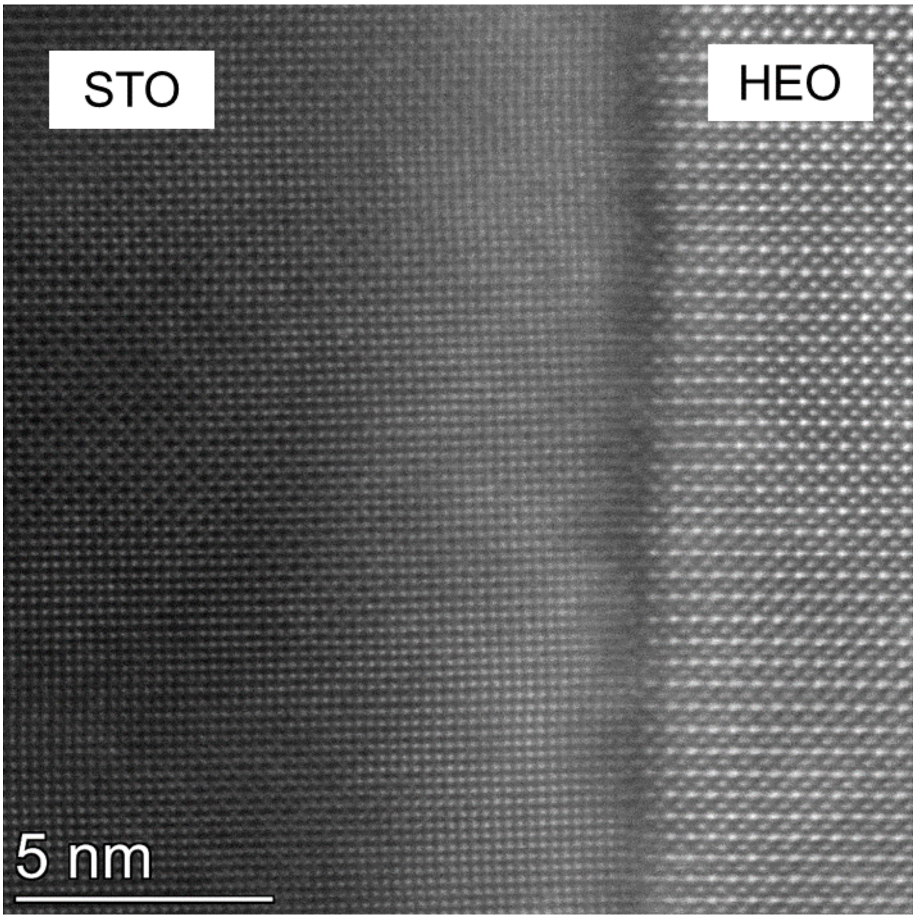

Figure S5. TEM micrograph of HEO (right) deposited on STO substrate (left). Lattice mismatch at the substrate-film interface could be observed, along with region of lattice continuity.

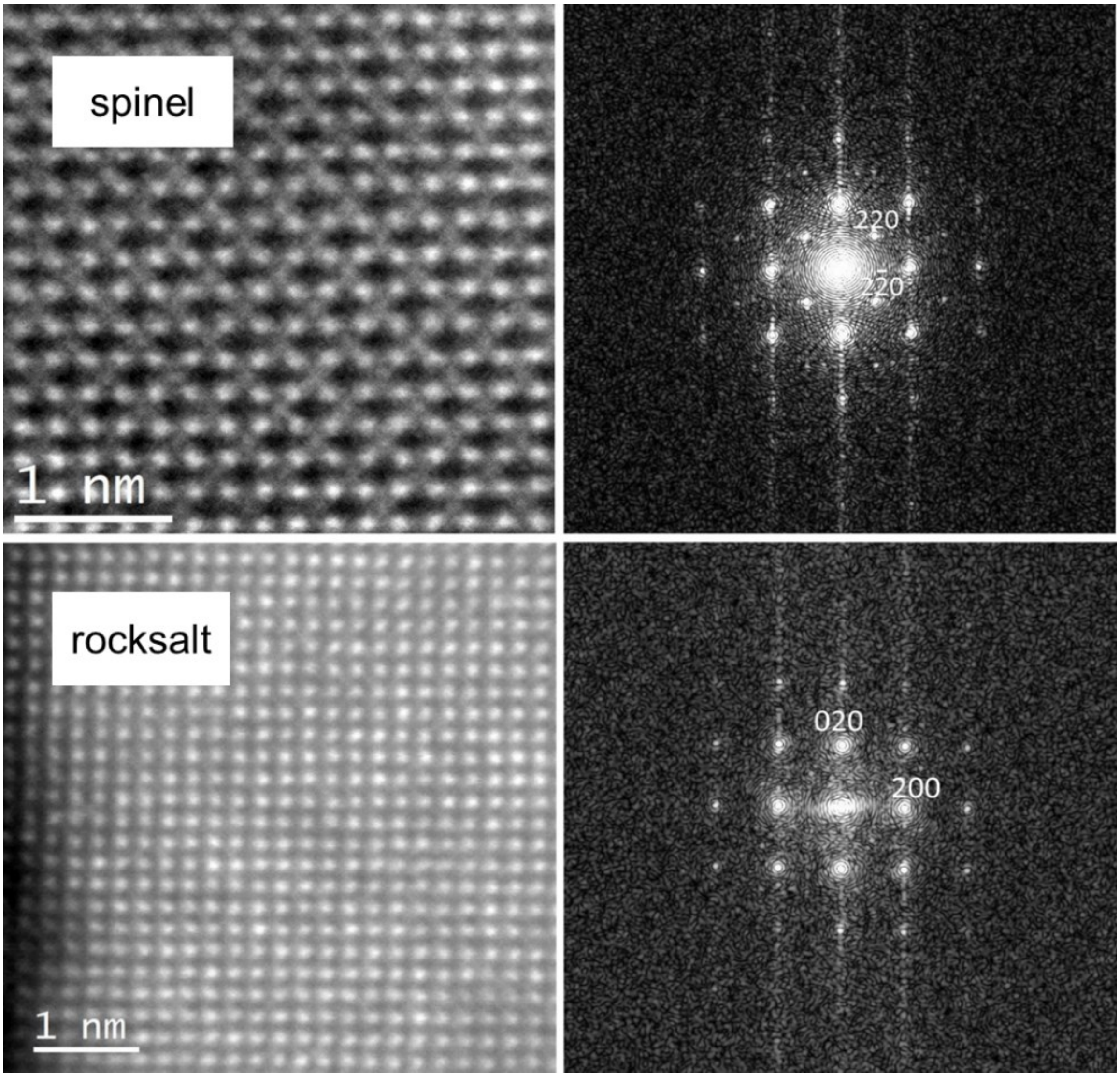

Figure S6. TEM micrograph along with corresponding FFTs of HEO deposited on STO. Coexistence of spinel and rocksalt phases is observed.

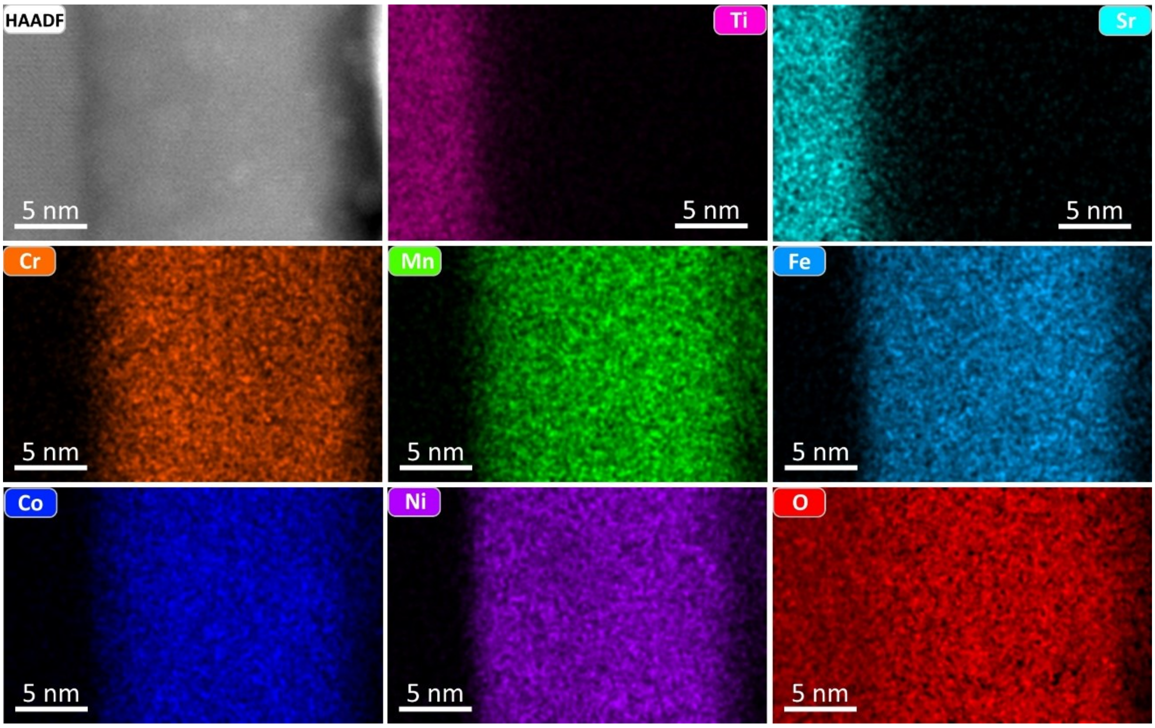

Figure S7. Elemental distribution maps of HEO on STO indicating no elemental segregation at the interior or interface of the HEO film.

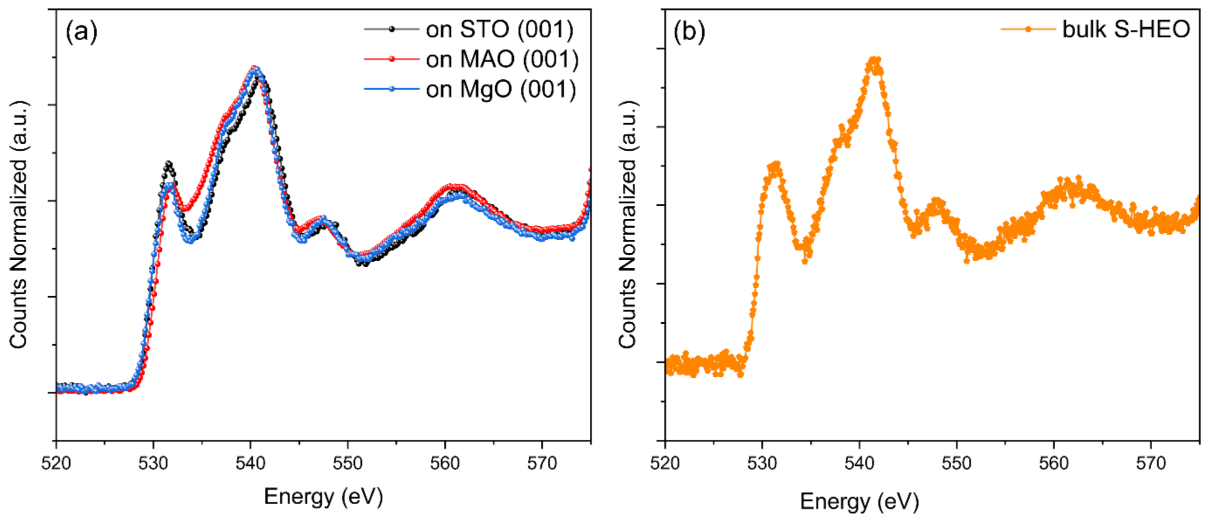

Figure S8. (a) Comparison of the O-K edge EELS data for HEO deposited on the three different substrates. The results indicate that spinel-HEO is the major phase, which is present in a similar amount in all the three cases. (b) Reference spectrum of bulk spinel-HEO.

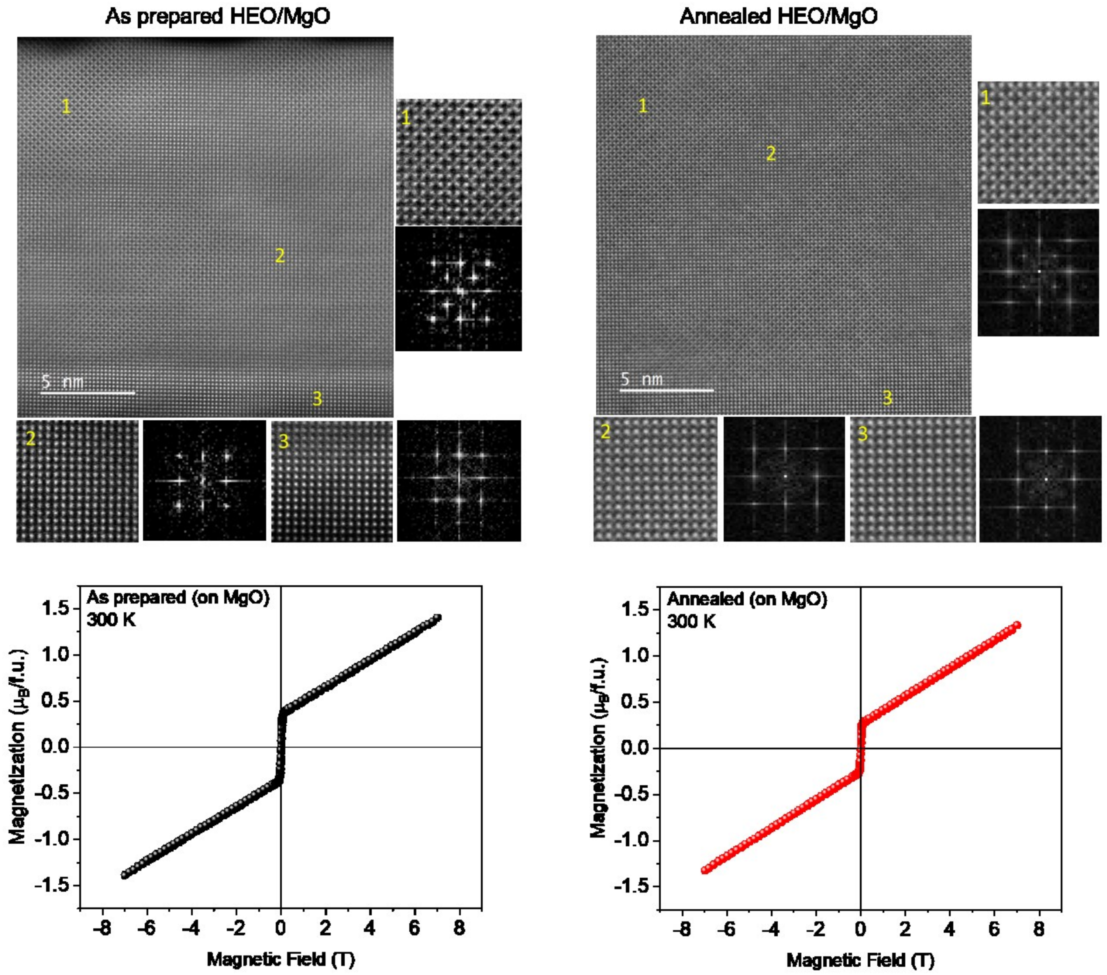

Figure S9. Cross-sectional HAADF-STEM images of as-prepared (left) and annealed (right) HEO films grown on MgO substrate. The regions 1, 2 and 3 correspond to the spinel-HEO phase, rocksalt-HEO phase and MgO substrate, respectively, as can be identified by the FFT patterns, which are observed in both as-prepared and the annealed samples.

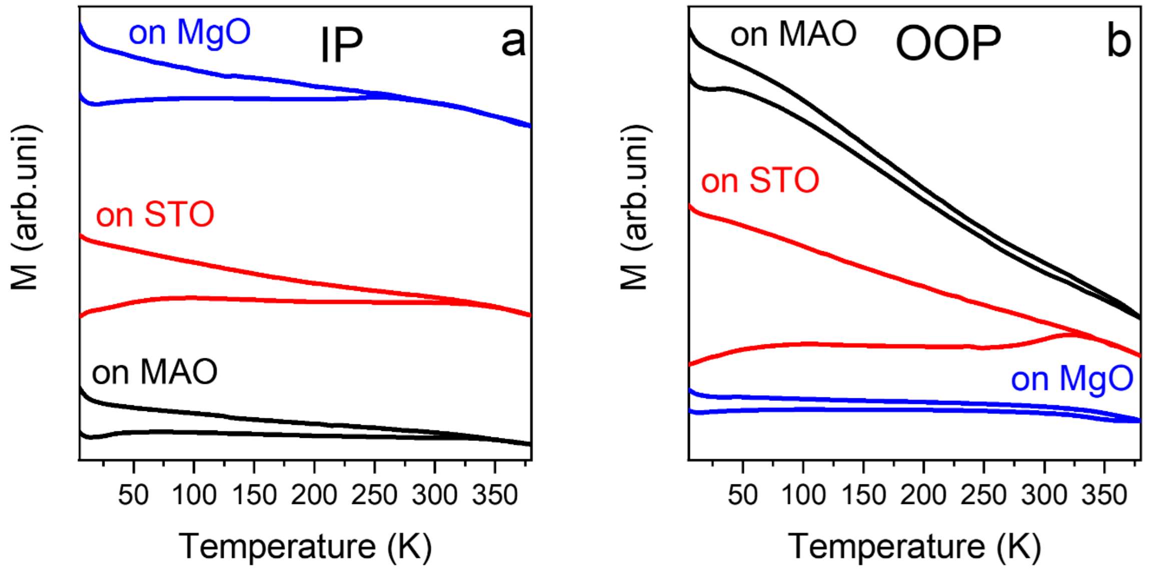

Figure S10. In-plane (a) and out-of-plane (b) *MT* curves of 20 nm HEO films deposited on different substrates measured at 500 Oe.

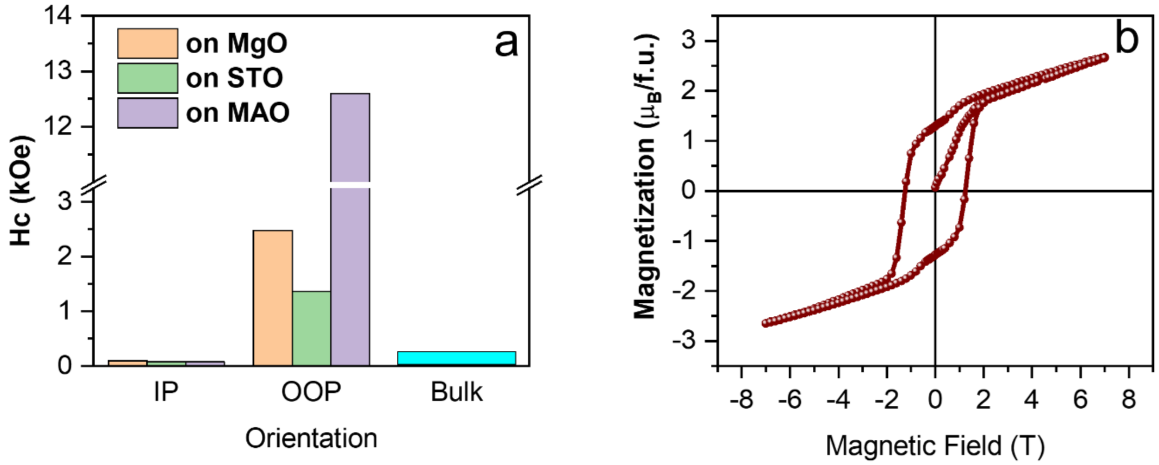

Figure S11. (a) Comparison of coercivity (H_c) among the HEO thin films and bulk HEO. (b) Virgin *MH* curve (out-of-plane) of 20 nm HEO on MAO at 5 K.

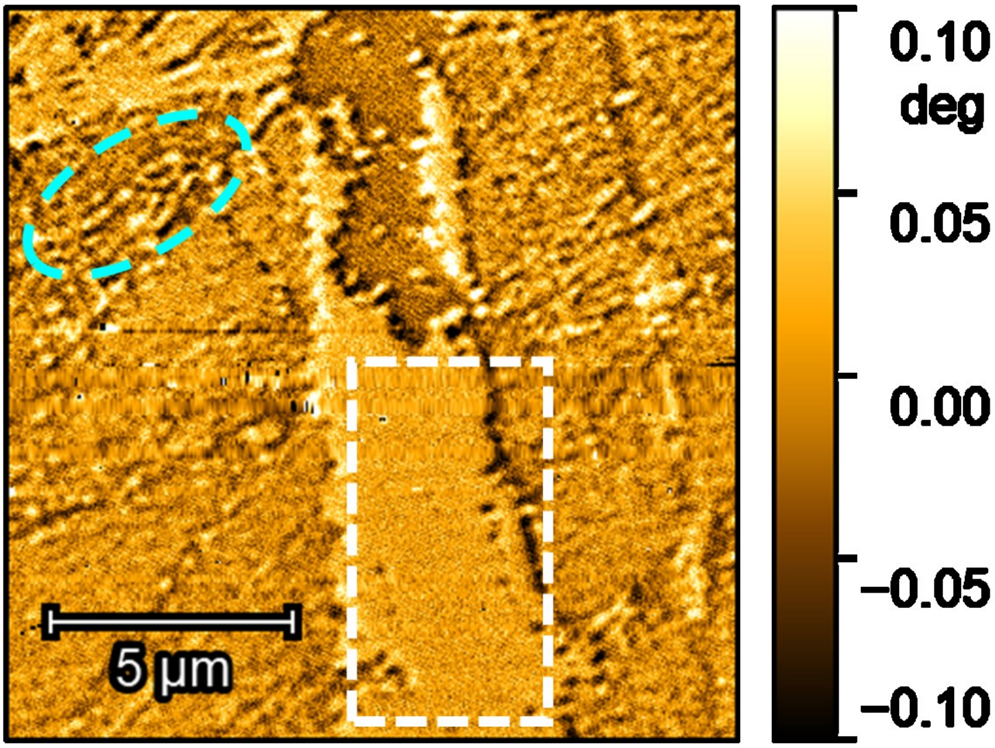

Figure S12. MFM of 20 nm HEO on MAO substrate. Cyan circle denotes the typical maze-like domain structure that highlights the perpendicular magnetic anisotropy, while the white rectangular area indicates the component with in-plane magnetization.

Ceramic target preparation. The powder precursor of $(Co_{0.2}Cr_{0.2}Fe_{0.2}Mn_{0.2}Ni_{0.2})_3O_4$ spinel was synthesized with the use of the modified Pechini sol-gel method. As starting chemicals, nitrates of all of the considered cations were used: $Co(NO_3)_2 \cdot 6H_2O$ (Alfa Aesar, 98-102%), $Cr(NO_3)_3 \cdot 9H_2O$ (Riedel-de Haën, ≥98%), $Fe(NO_3)_3 \cdot 9H_2O$ (Sigma Aldrich ≥98%), $Mn(NO_3)_2 \cdot 4H_2O$ (Alfa Aesar, 98%), and $Ni(NO_3)_2 \cdot 6H_2O$ (Alfa Aesar, 99.9985%). Citric acid monohydrate (Alfa Aesar 99.5+%) and ethylene glycol (EG) were used to respectively create the chelate of the cations and ensure polycondensation. The molar ratio of all the cations, citric acid, and EG in the mixture was 1:2:4. Initially, the nitrates were dissolved in demineralized water, and citric acid and EG were added. The mixture was then put on a magnetic stirrer, with heating plate set initially to 150 °C (esterification step). After obtaining a clear solution, the temperature was increased to 300 °C. The obtained gels were then calcined at 700 °C for 6 h, which was followed by a slow cooling (in the furnace), yielding fine, nanosized powders.
